# The Way of SARS-CoV-2 Pneumonia—An Early-Pandemic Review of the Key Manifestations and Severity

**DOI:** 10.3390/jcm14197096

**Published:** 2025-10-08

**Authors:** Dinko Bankov, Nedelina Kostadinova, Juliana Marinova

**Affiliations:** 1 Department of Propaedeutics of Internal Diseases and Clinical Laboratory, Faculty of Medicine, Trakia University, 6000 Stara Zagora, Bulgaria; dinko.bankov@trakia-uni.bg; 2 Department of Health Care, Medical College, Trakia University, 6000 Stara Zagora, Bulgaria; 3 Department of Social Medicine, Health Management and Disaster Medicine, Faculty of Medicine, Trakia University, 6000 Stara Zagora, Bulgaria; yuliyana.marinova@trakia-uni.bg

**Keywords:** COVID-19, pneumonia, infection, RNA virus

## Abstract

The disease COVID-19, which has befallen mankind in recent years, was a challenge that we had not faced for centuries. The first registered patient case was in China. This review is performed by the inspection of a large body of worldwide investigations conducted in the peak period of the disease’s progress. The disease is spread by airborne droplets and develops mainly with fever, cough, sputum, and shortness of breath. Laboratory tests show leukopenia, lymphopenia, a decrease in the levels of sodium, potassium, and calcium, and an increase in the levels of CRP, LDH, and D-dimer. Radiological changes in most cases are bilateral and of the “ground glass” type in the lower parts of the lungs. The most severe complication of COVID-19 pneumonia is ARDS. The risk groups are people with chronic lung diseases, the elderly, and those who are overweight. This article analyzes and summarizes the main characteristics of SARS-CoV-2 pneumonia in order to better understand and apply better clinical management of this condition.

## 1. Introduction

In Wuhan, China, on 30 December 2019, a bronchoalveolar lavage sample was taken from a patient with pneumonia. PCR testing revealed a betacoronavirus with a genome sequence identity of 96% to a bat coronavirus (BatCov RaTG13) [1], 79% to SARS-CoV, and 50% to MERS-CoV. The World Health Organization (WHO) has named this novel virus SARS-CoV-2, and the disease it causes is COVID-19 [2]. COVID-19 initially spread in China, and then it spread to several Asian and European countries, including Iran and Italy, where large outbreaks of infection developed, and the disease gradually spread worldwide [3]. On 11 March 2020, the WHO declared the occurrence of a pandemic.

SARS-CoV-2 is a positive-strand RNA virus. It has the same structure as other coronaviruses: membrane glycoprotein (M), envelope protein (E), spike protein (S), and nucleocapsid protein (N) [4].

This disease, COVID-19, that has befallen mankind in recent years, has been a challenge that has not been faced for centuries. The reasons for the difficulties that have arisen since the emergence of this new virus are the modern lifestyle and the lack of sufficient scientific knowledge about this disease. The objective of this literature review is to present the main characteristics of COVID-19 pneumonia—etiology, pathophysiology, epidemiology, clinical course, and laboratory and imaging studies in order to better understand and apply better clinical management of this condition.

## 2. Methods

PubMed and Google Scholar databases were used to perform a review of the existing studies on pneumonia description that were published during the pandemic’s peak period after its appearance.

Inclusion criteria: Investigations from the period 2020–2021 (these were the years of highest incidence and the highest levels of morbidity and mortality among people worldwide).

Exclusion criteria: Studies from 2022 and later were excluded; they would be more appropriate to summarize the long-term effects of the disease.

The terms used to search the literature were as follows: COVID-19 pneumonia, clinical, laboratory, and imaging characteristics of COVID-19 pneumonia, complications of COVID-19 pneumonia, respiratory failure in COVID-19, mortality in COVID-19 pneumonia, and risk groups in COVID-19 pneumonia. The research and selection process was carried out based on different types of original scientific literature that were openly accessible. These are described in Table 1.

## 3. Pathophysiology of COVID-19 Pneumonia

COVID-19 is spread by airborne droplets, after inhalation of aerosols released when talking, sneezing, or coughing in a 4 m radius, remaining temporarily in the air near a sick person, and it can also spread by the contact-household route, after touching infected surfaces [36]. The time that the particles released from the respiratory tract remain in the air depends on their size. At a particle diameter of 100 μm, they fall to the floor in about 10 s, at 10 μm diameter—17 min, and those with a diameter of 1 to 3 μm can remain in the air almost indefinitely. SARS-CoV-2 remains for up to 4 h on copper surfaces, 24 h on cardboard, and 2–3 days on plastic materials [41]. Then, the virus is transferred to the mucous membranes of the nose, mouth, or conjunctiva. Some scientists also suggest a fecal–oral route, since SARS-CoV-2 has been found in the gastrointestinal tract and in feces [36]. Studies by scientists show that the causative agent of COVID-19 uses the receptors for angiotensin-converting enzyme 2 (ACE2 receptors), just like SARS-CoV, to enter the host cell. These receptors are expressed in the kidneys, heart, and intestinal epithelial cells, but the lungs are the most affected. The explanation for this is that alveolar epithelial cells type two (AECII) are 83% of the cells expressing ACE2 receptors. The second reason is the large surface area of the lungs, through which the virus arrives [5]. The virus binds to the ACE2 receptor via the spike protein, but for this to occur, the cellular serine protease TMPRSS2 must cleave the spike protein into S1 and S2 parts [6]. The loss of infected alveolar epithelial type 2 cells leads to a decrease in surfactant production, which, in turn, leads to alveolar collapse [37]. In patients with COVID-19, damage to the endothelial layer of arteries and veins is observed, caused by the virus that has entered the cells via the ACE2 receptors. An inflammatory reaction occurs, and inflammatory cells accumulate—macrophages and neutrophils. The secretion of von Willebrand factor increases, leading to platelet adhesion to endothelial cells, hypercoagulability, and microthrombus formation [40].

The COVID-19 pneumonia develops in two stages. During the early stage, viral replication occurs, resulting in direct tissue damage, and during the late stage, an immune response is initiated. The pathogenesis of COVID-19 pneumonia is a complex process. It involves an inflammatory response and microvascular thrombosis induced by viral damage [48]. Neutrophils attempt to destroy the virus and release toxins and chemokines that further damage the alveoli [40]. A large immune response, called a “cytokine storm,” is formed, which also results in tissue damage [38]. Dendritic cells, macrophages, and monocytes accumulate at the site of damage, which secrete tumor necrosis factor alpha (TNF-α), tumor necrosis factor beta (TNF-β), ferritin, interleukin 1 (IL1), interleukin 10 (IL10), and interleukin 6 (IL6) [39]. These cytokines lead to the release of vascular endothelial factor (VEGF), and also IL6, IL8, and monocyte chemoattractant protein-1 (MCP-1). E-cadherin expression on endothelial cells is reduced, vasodilation occurs, capillaries become more permeable, and interstitial and alveolar edema occur. Fluid in the alveoli washes away surfactant, surface resistance increases, and the alveoli collapse, resulting in respiratory failure [48].

## 4. Clinical Characteristics of COVID-19 Pneumonia

The incubation period of SARS-CoV-2 is from 2 to 14 days, with an average of 3–5 days, and, in general, depends on the activity of the immune system [48]. The dose of virus ingested is also thought to be important [36]. A study measuring viral loads from nasopharyngeal secretions found that severe cases had about 60 times higher viral loads and were slower to clear the virus than milder cases. The main symptoms are fever in 83% to 99% of cases, cough between 59% and 82%, sputum in 28% to 33%, shortness of breath in 31% to 40%, fatigue in 44% to 77%, and muscle and joint pain in 11% to 35% [47]. Headache, sore throat, and runny nose may also be present [7]. In a prospective observational cohort study of 20,133 people of all ages in hospitals in the UK, the symptoms that were presented were as follows: fever in 71.6% of cases, shortness of breath in 71.2%, cough in 68.9%, and 4.5% of the patients were asymptomatic [26]. According to another retrospective, single-center case series of a study by the University Hospital in Wuhan, China, conducted among 138 patients, the median age was 56 years. In 75 men and 63 women, it was found that shortness of breath appeared within 1 to 10 days, 5 days on average, from the onset of the first symptoms, and hospitalization was between 4 and 8 days, 7 days on average, from the onset of the disease. The development of acute respiratory distress syndrome took from 6 to 12 days, 8 days on average [11].

According to some studies, pneumonia is confirmed by X-ray within 3 to 9 days of the onset of the first symptoms, and admission to the intensive care unit occurs within 6 to 12 days [8]. The time from the onset of the disease to mechanical ventilation is 14.5 days, and to lethal outcome 18.5 days [10].

Based on data from a call study involving 1487 patients in Paris, France, with an average age of 44 years, and 700 men and 752 women, the following was found: fever and dry cough were observed in 91% of the cases, asthenia in 60%, muscle pain in 57%, headache in 55%, shortness of breath in 32%, chest pain in 22%, loss of taste and smell in 23%, and hemoptysis in 3% [9]. In 11.6% of the subjects studied, impaired olfaction was the first symptom. Complete loss of smell was reported in 67.6% of the patients and partial reduction in olfaction in 32.4%, while 59.2% complained of taste disturbances [29].

A meta-analysis of seven studies involving 1813 patients was conducted, median age of 62.4, men 67.2% and women 32.8%, according to which patients can be divided into two groups: the first group includes patients in serious condition, and the second one includes patients in intensive care. The characteristic symptoms for those in serious condition are cough in 70.5% of the cases, fever in 64.1%, and fatigue in 44.5%. In the second group, the main symptoms are cough in 67.2%, fever in 62.9%, and shortness of breath in 61.2%. Shortness of breath is the symptom that determines the severity of the course and admission to the intensive care unit [12]. According to some doctors, a high respiratory rate is one of the indicators of a fatal outcome [46]. In conclusion, we can say that the main clinical manifestations are cough, sputum, fever, fatigue, and shortness of breath. The high frequency of complaints of shortness of breath, in contrast to pneumonia with another cause, is striking. So, in cases where we have a patient with pneumonia and shortness of breath, we should always consider that the possible causative agent is SARS-CoV-2.

## 5. Laboratory Characteristics

Innate immunity is the first defense response of the human body against pathogens. The cells involved are macrophages, neutrophils, NK cells, and dendritic cells. They recognize micro-organisms and destroy them [48]. A study conducted among 326 patients found a decrease in CD3+, CD4+, and CD8+ T lymphocytes, with CD3+ being the most affected. IL6 and IL8 cytokines were increased, which were inversely correlated with the number of lymphocytes [13]. From the studies of 103 patients in a hospital in Nanchang, China, a significant decrease in CD3+, CD4+, and CD8+ T lymphocytes and NK cells was observed, and in more severe cases, this decrease was significantly more pronounced. The ratio between CD4/CD8 is increased, i.e., CD4 T lymphocytes are less than CD8, and this is also indicated as a sign of a more severe course. In this study, all patients in severe conditions recovered. They had the greatest decrease in T-cells during the first week, and in the second week, the cells began to increase in number, and in the third week, they had values similar to those who had a milder course of the disease. After the PCR test for SARS-CoV-2 was negative, T-lymphocytes began to rapidly reach their normal number. The authors pointed out the possibility that the T-lymphocyte count could serve as a prognostic marker for the severity of the course [14]. In a study of 27 mild and 13 severe cases, the data showed lymphopenia in 44.4% of the mild and 84.6% of the severe cases. The lowest lymphocyte levels in severely ill patients were found around day 4–6, and after 7 to 15 days, the levels started to rise. Mainly, CD3+, CD8+, and CD4+ T-cells were reduced. Serum levels of IL6 and IL10, and IL2 and IFN γ cytokines were increased in severe cases. The IL6 level started to decrease on day 16, and that of IL10 on day 13 from the onset of symptoms, while IL2 and IFN γ showed a peak on day 4–6. Prognostic factors for detecting severe cases may be the ratio of neutrophils to CD8+ T-cells and of neutrophils to lymphocytes [33]. An analysis of 31 articles examining a total of 46,959 patients showed a decrease in lymphocytes (lymphocytopenia) in 57.4% of the cases, a decrease in leukocytes (leukocytopenia) in 36.9%, and increased leukocytes (leukocytosis) in 11% of the cases, and an increased CRP level in 61.3%, an increased lactate dehydrogenase (LDH) in 57%, and an increased erythrocyte sedimentation rate (ESR) in 42.2% [42].

An analysis of five studies involving 1415 people showed decreased serum sodium, potassium, and calcium levels in patients with severe COVID-19. Scientists suggest that the reduction in ACE2 after its binding to the virus increases angiotensin II, which leads to increased renal excretion of potassium. Other causes of electrolyte disturbances can be diarrhea and vomiting. Of concern is hypokalemia, which is known to exacerbate the acute respiratory distress syndrome and lead to cardiac disorders [34].

D-dimer and CRP were studied in 577 patients, of whom 144 died. Physicians concluded that higher levels and faster increases in these markers were observed in those who died [16]. In addition, another study indicated that a more severe course of the disease occurred when CRP was above 41.8 mg/L, IL6 was above 32.1 pg/mL, and procalcitonin was above 0.07 ng/mL [17]. More severe cases of COVID-19 had elevated inflammatory markers characteristic of bacterial infection, such as CRP, procalcitonin, ferritin, and eosinopenia. It is noteworthy that the increase in IL6 correlates with the increase in CRP [39]. In patients in the intensive care unit, an increase in D-dimer and prothrombin time (PT) was reported. In this study, the D-dimer of the ICU patients ranged from 0.6 to 14.4 mg/L, and the PT was from 11.2 to 13.4 s, while the remaining patients had D-dimer from 0.3 to 0.8 mg/L and PT from 9.8 to 12.1 s. Upon admission, 69% of the patients had normal procalcitonin <0.1 ng/mL, and plasma concentrations of IL7, IL8, IL9, IL 10, IL1B, IL1RA, IFN γ, TNF α, FGF, GCSF, GMCSF, PDGF, MCP1, MIP1A, MIP1B, and VEGF were higher. Subsequently, higher concentrations of IL2, IL7, IL 10, TNF α, GCSF, MCP1, MIP1A, and IP10 were reported in the ICU patients [15].

In severe cases, troponin levels were elevated. This was found in a study of 2736 people in New York City, which found elevated levels in 985 of them [3].

Of the 319 patients studied, 64 of whom died, lower albumin levels were found in the deceased. It is suggested that an albumin level below 32 is predictive of a fatal outcome [35].

The main laboratory characteristics that distinguish COVID-19 pneumonia are lymphocytopenia, leukocytopenia, increased D-dimer and prothrombin time (PT), and decreased serum sodium, potassium, and calcium levels, suggesting a more severe course. CRP, ESR, and LDH levels are also elevated, which is characteristic of inflammatory diseases. If a combination of several of these changes is present, the clinician should consider possible COVID-19 pneumonia. Table 2 presents a summary of the biochemical markers in patients with COVID-19 pneumonia. The table contains trends in biochemical disorders and comments on particular cases.

## 6. Imaging Characteristics of COVID-19 Pneumonia

An analysis of 31 articles, including 46,959 patients, showed bilateral pneumonia in 75.5% of the cases and unilateral pneumonia in 20.4%. The changes were of the ground-glass type in 69.9%, halo sign in 54.4%, air bronchogram in 51.3%, thickened bronchovascular bundle in 39.5%, reticular shadow in 24.4%, and hydrothorax in 18.5% [42].

### 6.1. X-Ray of Lungs

Early in the course of COVID-19, and in milder cases, X-rays may not show any changes. As the disease progresses, ground-glass changes begin to appear [48].

X-rays of 234 people with COVID-19 showed bilateral involvement in 69.2%, most commonly basal in 58.5% and peripheral in 57.7%. Reticulo-nodular opacities were observed in 66.6%, ground-glass changes in 62.8%, consolidation in 57.7%, nodules in 23.5%, vascular congestion in 39.3%, and pleural effusion in 16.6% [20]. A study in Italy of 260 patients with COVID-19 who had X-rays showed radiological changes in 159 of them, and no such changes were found in 101. In patients with changes, it is characteristic that the X-ray image was taken at least 4 days after the onset of symptoms, and there was an increase in LDH above 500 UI/L and CRP above 30 mg/L. When all three factors were present, 95.3% of the patients had a radiological finding, 89.2% in the presence of two factors, and 40.7% in the presence of one factor [21]. A large-scale study conducted in the Lombardy region, Northern Italy, including 1171 patients with COVID-19 aged 18 to 96 years, with an average age of 63.3 years, showed that in most patients the disease proceeded with complaints of high fever in 79.9%, cough in 50.7%, and without symptoms in 4.6%. Radiological changes were present in 80.3%, i.e., 940 patients, whose average age was 65 years, and among those with normal X-rays, it was 56.6 years. Changes were bilateral in 73.9%, peripheral in 55.8%, and in the mid-lower zone in 33.1%. The most common radiological finding was ground-glass opacities in 621 patients, reticular pattern in 426, consolidation in 224, and pleural effusion in 7. After an average of 4 days, a second image was taken in 382, of which 35 had changes as early as the first image, while for 31 people with a normal first image, on the second one, 26 of them had changes. Deterioration of the image was observed in 68.6%, no significant changes were observed in 19.1%, and resorption of infiltrates in 12.3%. Of these 382 patients, 59.4% had ground-glass opacities on the first image, while the percentage decreased to 51% on the second image, while the reticular pattern also decreased from 42.4% to 37.2%. The increase in consolidation is striking, with consolidation being detected in 26% on the first image and in 53.4% on the second image. However, consolidation was not present in 116 patients on the first image, while it was detected on the second one [22].

It can be concluded that COVID-19 pneumonia is most often bilateral, and the radiological changes are of the reticulo-nodular opacities, ground-glass opacities, and consolidation types. Radiological changes were observed in patients with symptoms present for at least 4 days and elevated inflammatory markers from laboratory tests. In these cases, we recommend that the patient be tested for SARS-CoV-2.

### 6.2. Computed Tomography of Lungs

Five stages are distinguished in the computed tomography (CT) image of the lungs in COVID-19. During the first stage, in asymptomatic patients, single or more scattered foci of ground-glass opacities are seen, uneven consolidation with air bronchogram, and nodules in the center of the lobule surrounded by ground-glass opacities. The second stage occurs one to three days after the onset of symptoms. Then the capillaries in the alveolar septa are filled with blood, interstitial edema is observed, and exudate pours into the alveoli. It presents as single or multiple scattered ground-glass opacities, separated like a honeycomb by thickened interlobular septa. After three to seven days from the onset of symptoms, interstitial and alveolar edema increase, and the exudate is rich in cells. A large infiltrate is observed, which consolidates with the presence of a bronchogram. The next stage, which occurs seven to fourteen days after the onset of symptoms, is characterized by fibrous exudation. The image is represented by numerous irregular consolidations of smaller areas and density. During the final, fifth stage, covering the period from two to three weeks after the clinical manifestation, changes decrease, consolidations are irregular, and the interlobular septa are thickened [23]. Lung scans of 63 patients with COVID-19 showed involvement of only one lobe in 30.2% of the cases, involvement of two lobes in 7.9%, involvement of three lobes in 6.3%, involvement of four lobes in 11.1%, and involvement of five lobes in 44.4%. Changes were ground-glass punctate in 85.7% of cases, ground-glass nodules in 22.2%, patchy consolidation in 19.0%, fibrous stripes in 17.5%, and heterogeneous solid nodules in 12.7%. Progression in imaging findings was observed in 85.7% of patients [24].

A study looked at two groups of patients, one with 58 patients in better condition and the other with 25 in severe condition. Ground-glass focal lesions were found in 97.6% of all patients, linear opacities in 65.1%, consolidation in 63.9%, interlobular septal thickening in 62.7%, and the crazy-paving model in 36.1%. Consolidation, crazy-paving, linear opacities, and interlobular septal thickening were more common in those with a severe condition. Pericardial effusion, pleural effusion, and enlarged lymph nodes were also observed. Multiple lobes were also affected [28]. Data from CT scans made on 24 patients with COVID-19 found that the most common locations of lung lesions were the subpleural, lower, and posterior lung fields [19]. In another study of 101 patients with COVID-19, CT data showed bilateral changes in 82.2% and mostly in the lower lung lobes in 54.5% [30].

A pulmonary CT angiogram was performed on 106 patients with COVID-19, and 32 (30%) of them had pulmonary embolism, with elevated D-dimer levels [31]. Another study performed pulmonary CT angiography on 55 patients with COVID-19. In this study, pulmonary vascular thrombosis was detected in 28 patients, multiple in 22, and bilateral in 16. The right lung was the most affected, with involvement in the lower right lobe in 18 patients, the upper right lobe in 17, and the middle right lobe in 8 patients, while the left lung was affected in the lower left lobe in 17 patients and the upper left lobe in 13 patients. The affected arteries were lobar in 6 of the 28 patients diagnosed with thrombosis, segmental in 19, and subsegmental in 27. Vessels were dilated at the subsegmental level in 31 patients, with a diameter of 4.42 mm to 6.4 mm in those with thrombosis and 2.55 mm to 3.2 mm in those without thrombosis. The D-dimer level in those with thrombosis was 1.78 µg/mL to 21.00 µg/mL, and from 0.93 µg/mL to 6.06 µg/mL in those without thrombosis [27].

Lung CT scans revealed bilateral localization of infiltrative changes, mainly affecting the lower lung fields subpleurally, with several lobes of the lung involved. Contrast-enhanced studies showed a high incidence of pulmonary embolism.

## 7. Respiratory Failure

A systemic inflammatory response resulting from direct or indirect lung damage can lead to acute respiratory distress syndrome (ARDS). Epithelial and endothelial cell destruction resulting in diffuse alveolar damage with exudation is the presumed cause of ARDS in COVID-19 [45]. Respiratory failure that develops in COVID-19 pneumonia is due to several mechanisms: hypoventilation, impaired diffusion, shunting, and impaired ventilation/perfusion ratio, occurring in areas where ventilation is reduced or absent, and blood flows through there. Increased thrombus formation, leading to microembolism, also leads to reduced perfusion in the affected area. Two phenotypes have been distinguished: acute respiratory distress syndrome type L with high compliance and reduced ventilation/perfusion ratio, and type H with low compliance and large right-to-left shunt [44]. Signs of developing ARDS include hypoxia unresponsive to oxygen therapy, as well as tachypnea, tachycardia, and hypotension [48]. The mortality rate for ARDS due to COVID-19 ranges from 26% to 61.5%. This is higher than for other etiologies, where it ranges from 35.3% to 40.0% [32].

The most severe complication of COVID-19 pneumonia is the development of ARDS with respiratory failure. The mechanisms that cause it are hypoventilation, impaired diffusion, impaired perfusion due to microembolism, and shunting, and the result is a disorder in the ventilation/perfusion ratio.

## 8. Risk Groups

Older people and those with comorbidities are susceptible to a more severe course. In children, the development of pneumonia, increased inflammatory markers, and lymphocytopenia are very rare, and the incidence in children is 1% to 5% of the total. It is assumed that the reason for this is the lower expression of ACE2 and TMPRSS2 in children in the upper and lower respiratory tracts, which has been found in studies. In adult smokers with chronic obstructive pulmonary disease (COPD) and hypertension, the expression of ACE2 and TMPRSS2 is significantly higher. People with bronchial asthma have a higher expression of TMPRSS2 in the bronchial epithelium, but not of ACE2, compared to healthy people, which predisposes them to a more severe course [43]. A study was conducted, including 78,674 patients, in which 26.8% of them, i.e., 20,196, had a comorbidity of bronchial asthma or another lung disease, or both. There were 860 patients under the age of 16, of whom 8.6% had asthma, and there were no significant clinical differences between patients with and without asthma. There were 8950 patients aged 16 to 49, of whom 20.9% had asthma. There were 65,653 patients over the age of 50, of whom 9.0% had asthma, 6.0% had another chronic lung disease, and 3.2% had asthma overlapping with another chronic lung disease. The prevalence of bronchial asthma among the general population was about 7% for each age group [18].

In the United States, 42,604 patients admitted to the hospital with COVID-19 were included in a study. The proportion of people over 65 years of age was the highest, 46.8%, and then from 50 to 64 years of age, 27.2%, and from 18 to 49 years of age, 24.9%, and there were 454 cases of those under 18 years of age. The mortality rate among people over 75 years of age was 20.9%, and in the distribution between men and women over 75 years of age, by gender, the mortality rate in men was higher at 12.5%, and in women at 9.6% [25].

Patients at risk for a more severe course of the disease are those with chronic lung diseases such as bronchial asthma and COPD, and other predisposing factors are age, especially patients over 65 years of age, as well as obesity. For these risk groups, prophylaxis should be easily accessible, including vaccination and immunostimulation, and consideration should be given to possible infection with SARS-CoV-2 in the presence of a respiratory infection.

## 9. Limitations and Future Directions

The limitations of the article are expressed as follows: Numerical expression of specific laboratory values, therapy, and follow-up after illness. The methodology we use does not include analysis of our own data or comparisons of specific numerical values from other studies, but the expression of trends. The reason why an analysis of the response after applied therapy and consequences after illness is not included is that we aimed to focus the readers’ attention on pathophysiology, clinical characteristics, laboratory characteristics, imaging diagnostics, respiratory failure, and risk groups.

Future directions would be connected to a presentation of our own data with specific numerical data of biochemical markers, clinical characteristics, and imaging diagnostics, and their change after a specific applied treatment, and identification of long-term consequences after illness.

## 10. Conclusions

COVID-19 pneumonia is a serious disease that requires increased attention in diagnosis and, subsequently, in treatment. In an attempt to find an appropriate treatment and vaccine, numerous studies were conducted around the world to better understand this infection. It manifests clinically with cough, sputum, fever, and high frequency of shortness of breath. From laboratory tests, we observe lymphocytopenia, leukocytopenia, increased D-dimer, CRP, and LDH, and decreased sodium, potassium, and calcium, as was mentioned earlier in the review during discussion of more severe cases. From lung imaging tests, radiography, and CT scan, it is found that in most cases there are bilateral changes, mainly in the lower lung sections, subpleurally, affecting several lobes. The most severe complication is ARDS, occurring with respiratory failure. A higher risk for a more severe course of the disease is observed in male patients, over 65 years of age, with chronic lung diseases such as bronchial asthma and COPD, and obesity. The combination of most of these indicators in a patient should prompt clinicians to test them for COVID-19. Early diagnosis of the etiology of the cause of pneumonia will provide a greater chance of rapid and successful treatment without complications or with minor ones.

This article on COVID-19 pneumonia reviews the major characteristics of this disease (etiology, pathophysiology, epidemiology, clinical course, laboratory, and imaging tests). This summary analysis aims to provide a clear understanding and diagnosis of this disease. In the future, it would be beneficial, in the event of a new respiratory pandemic during the early period before a vaccine is discovered, to have an etiological treatment available or herd immunity established.

## Figures and Tables

**Table 1:** Presentation of the literature employed by type, author, and year.

Type of Article	First Author	Year of Publication
Original research paper	Sun L. [4]	2021
	Zhang H. [5]	2020
	Hoffmann M. [6]	2020
	Chen N. [7]	2020
	Yang X. [8]	2020
	Lapostolle F. [9]	2020
	Zhou F. [10]	2020
	Wang D. [11]	2021
	Jain V. [12]	2020
	Zhang X. [13]	2020
	Jiang M. [14]	2019
	Huang C. [15]	2020
	Valerio L. [16]	2021
	Liu F. [17]	2020
	Bloom CI. [18]	2021
	Zhao S. [19]	2021
	Cozzi D. [20]	2020
	Gatti M. [21]	2020
	Vespro V. [22]	2021
	Jin YH. [23]	2020
	Pan Y. [24]	2020
	Finelli L. [25]	2021
	Docherty A.B. [26]	2020
	De Cobelli F [27]	2021
	Li K. [28]	2020
	Barillari MR. [29]	2021
	Zhao W. [30]	2020
	Léonard-Lorant I. [31]	2020
	Gibson PG. [32]	2020
	Liu J. [33] Lippi, G. [34] Violi, F. [35]	2020 2020 2021
	Dhama K. [2]	2020
	Guo G. [36]	2020
	Choi HM. [37]	2021
	Varghese PM. [38]	2020
	Root-Bernstein R. [39]	2021
	Cascella M. [40]	2021
	Dhand R. [41]	2020
	Cao Y. [42]	2020
	Singh R. [43]	2021
	Allado E. [44]	2021
	Li X. [45]	2020
Correspondence	Wang Y. [46]	2020
	Liu Y. [47]	2020
Report	WHO Situation Report [1]	2020
Book	Kamps B. and Hofmann C. [3]	2021
	Kriz C., Imam N., Zaidi S. [48]	2020
	Hoffmann C., Kamps B. S. [6]	2021

Source: Personal presentation.

**Table 2:** A summary presentation of biomarker changes in COVID-19 pneumonia.

Biomarkers	Biomarker Abnormality	Comment
**CD3+, CD4+, and CD8+ T lymphocytes**	decreased	In more severe cases, the decrease is more significant.
**NK cells**	decreased	In more severe cases, the decrease is more significant.
**The ratio between CD4/CD8**	increased	Indicated as a sign of a more severe course.
**IL6, IL8 и IL10, IL2 and IFN γ**	increased	In more severe cases, the increase is more significant.
**CRP**	increased	A more severe course of the disease occurs when CRP is above 41.8 mg/L.
**LDH**	increased	In more severe cases, the decrease is more significant.
**Sedimentation rate of erythrocytes (ESR)**	increased	In more severe cases, the decrease is more significant.
**Serum sodium level**	decreased	In more severe cases, the decrease is more significant.
**Serum potassium level**	decreased	In more severe cases, the decrease is more significant.
**Serum calcium level**	decreased	In more severe cases, the decrease is more significant.
**Procalcitonin**	decreased	In more severe cases, there is an increase.
**Ferritin**	increased	In more severe cases, there is an increase.
**D-dimer**	increased	In more severe cases, the increase is more significant.
**Prothrombin time (PT)**	increased	In more severe cases, the increase is more significant.
**Troponin**	increased	In severe cases, troponin levels increase.
**Albumin**	decreased	Below 32 g has prognostic value for the occurrence of a fatal outcome.

Source: Personal presentation.

## Data Availability

No new data were created or analyzed in this study.

## Abbreviations

The following abbreviations are used in this manuscript:

ACE2	Angiotensin-converting enzyme 2
ARDS	Acute respiratory distress syndrome
COPD	Chronic obstructive pulmonary disease
COVID	Coronavirus disease
CRP	C-reactive protein
CT	computed tomography
ESR	erythrocyte sedimentation rate
LDH	lactate dehydrogenase
WHO	World Health Organization
